# Methodology in specimen fabrication for *in vitro* dental studies: Standardization of extracted tooth preparation

**DOI:** 10.4317/jced.54020

**Published:** 2017-07-01

**Authors:** Lucía Fernández-Estevan, Diego Millan-Martínez, Antonio Fons-Font, Rubén Agustín-Panadero, Juan-Luis Román-Rodríguez

**Affiliations:** 1 Doctor in Dentistry (DDS; PhD). Master Buccofacial Prosthetics (M.Sc). Associate Lecturer, Department of Dental medicine, Prosthodontic and Occlusion Teaching Unit, UVGS, Spain; 2 Lecturer in Prosthodontics, UVGS, Spain; 3 Doctor in Dentistry (DDS; PhD). Assistant Lecturer, Department of Dental medicine, Prosthodontic and Occlusion Teaching Unit, UVGS, Spain

## Abstract

**Background:**

Specimen preparation for *in vitro* study suffers a general lack of methodological homogeneity, as well as a lack of uniformity in the materials selected for fabricating them. This situation prevents comparisons between studies. When a specimen is not of dental origin it is not possible to study adhesion or bonding techniques realistically. The objective is to design and implement a method of specimen preparation that permits universal standardization for use in *in vitro* studies.

**Material and Methods:**

A metal stump of specified dimensions was designed and fabricated by hand. It was scanned, the data digitalized, perfecting and standardizing the dimensions. Ten human molars were adapted to the size and shape of a standard milling block. A Cerec 3D inLab Cerec milling unit was used to prepare the molars to match the digitalized model.

**Results:**

Ten specimens with identical dimensions were obtained.

**Conclusions:**

CAD-CAM technology allows the preparation of natural extracted teeth to be standardized and could be used to establish a reproducible method that would facilitate comparison between different *in vitro* studies, and reduce bias arising from variations in sample fabrication.

** Key words:**Test ceramic, dentin analog, dentin model, dentin samples, methodology.

## Introduction

One of the main problems facing *in vitro* research in the field of dentistry is the lack of homogeneity in methodology (1), a situation that limits the application of the concepts of evidence based dentistry. It also prevents exact comparisons between different works of research, even when the same materials and techniques are used, and impedes extrapolation of the results obtained (2).

Most *in vitro* trials of new materials focus on the fabricated samples or restorations but pay little attention to the characteristics of the tooth stump to which the restorations are to be bonded (1). There are various methods for fabricating specimens by hand; all aim to be comparable within the study underway but the lack of standardization prevents extrapolation of the results to other centers or research teams who may wish to reproduce or develop the research (3). This does not favor the repetition of a study by another team that wishes to verify and contrast the data obtained, enlarge the sample size, or vary the bond materials used. Another problem is that these hand-made stump specimens are made from different materials (epoxy resins, different noble metals or alloys, polymethylmethacrylate, or, glass-fiber-filled epoxy) (1,3-8), which do not always behave in the same way as a natural tooth. Some do not admit current bond techniques.

CAD-CAM technology allows the design and fabrication of dental restorations by means of computer-assisted mechanization, using design software to control automated milling (9). This technology standardizes the design and fabrication of samples, whether they are discs, bars, cylinders, or restorations, fabricating identical specimens for the *in vitro* assay of restoration materials, cements or bonding techniques. In addition to the standardization of samples, an attractive feature of these technologies is that they may be applied to specimens of dental origin. Using dental specimens that have been digitally standardized would greatly facilitate comparison of the data obtained between studies and the extrapolation of the results to other research situations.

The objective is to design and implement a method for preparing dental specimens that would permit universal standardization in *in vitro* trials. To do this, the study set out to obtain ten specimens of dental origin that would be exact replicas of a computer-generated design 

## Material and Methods

**Figure 1:**
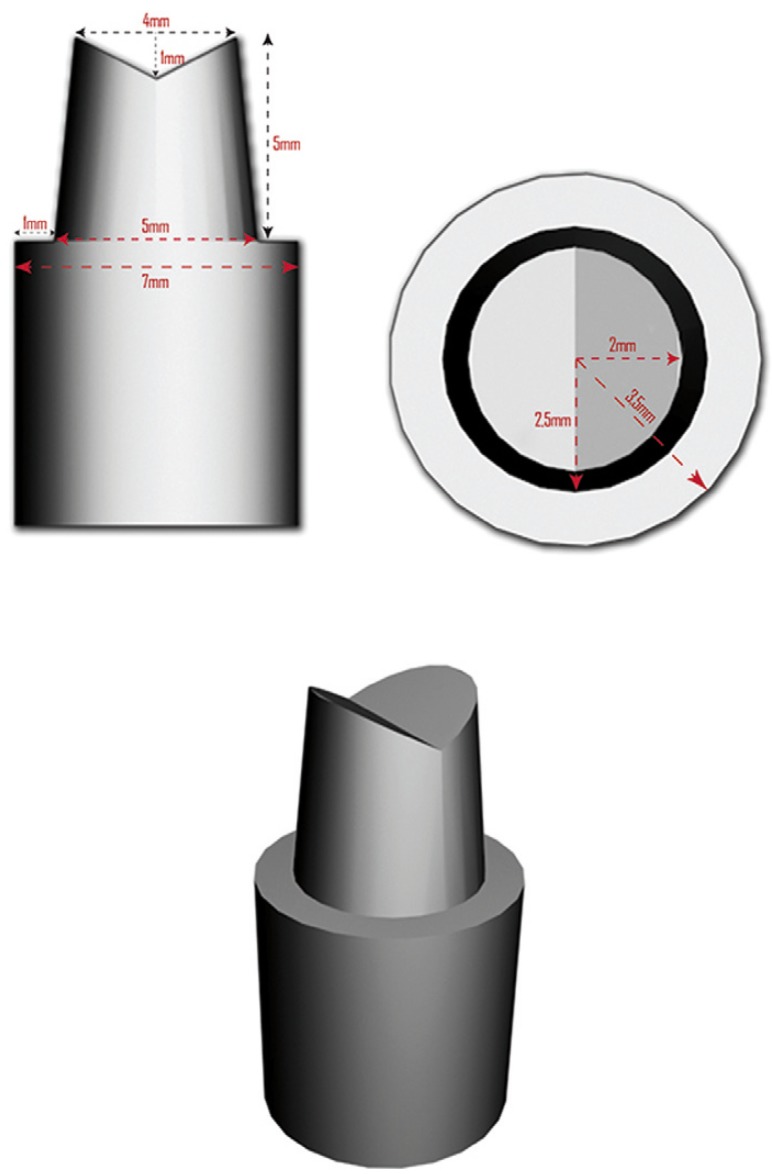
3D design of master model.

Standardization commenced with the elaboration of a hand-made metal stump, so a plan and profile of specific dimensions was created (Fig. 1), with the following features.

• Height (5mm), occlusal diameter (4mm), and base height (7mm).

• Occlusal notch with a depth of 1mm to simulate the cuspids and prevent crowns from rotating on the stump.

• 6o convergence of the axial walls. Rounded occlusal-gingival angles.

• Finishing line: chamfer with a thickness of 1 mm with rounded inner corner, and an axis-cervical angle of 120o.

An impression of metal stump was taken with high-precision addition silicon (Eurovipi Duplicad®, Eurovipi, Madrid, Spain) and then cast in Type IV plaster (IV Kimberlit®, Protechno, Girona, Spain). The plaster duplicate was laser-scanned using a CAD/CAM milling system (Sirona® CEREC 3D inLab, Sirona Dental Systems Gmbh, Bensheim, Germany), with approximately 5μm measurement precision and 0º, +45º angulation. When the specimen has been scanned, the digital image appears on the computer screen. Software tools can be used to modify and perfect the structure, trimming, smoothing, etc. In this way, the design and specifications of the virtual stump were brought to completion, before proceeding to its reproduction.

**Figure 2:**
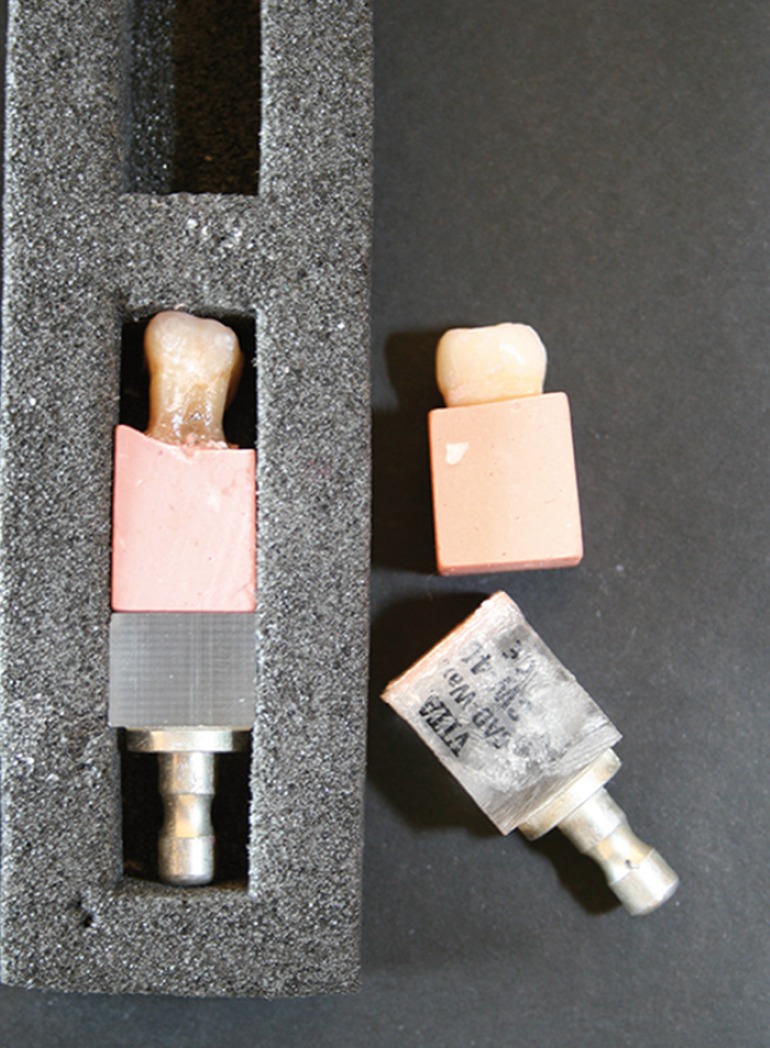
Molded packaging used as a pattern form managing block length.

Fabrication of the dental specimens began by selecting ten upper and lower first molars, extracted in response to periodontal problems, without caries or previous restoration; these were conserved in 0.5% chloramines at 4o for no longer than 90 days. The occlusal surfaces were smoothed down in order to reduce irregularities deriving from cusps and grooves. These were set in type IV plaster (Vel-Mix Stone®, Kerr S.r.l. Scafati, Italy) in a square mold, placing them so that the occlusal surface was horizontal. Using the molded milling block packaging, each specimen was adapted to the height of a standard block of VITA CAD-Waxx® CW-40 (VITA, Bad Säckingen, Germany) (Fig. 2), attaching the plaster base bearing the molar to the lower part of an original block of the milling material, fixing them in place with fast-setting adhesive (Renfert®, Hilzingen, Germany). The base part of the original block was then fixed to the Cerec milling machine’s attachment platform, which could be identified by the milling system’s calibration feature.

**Figure 3:**
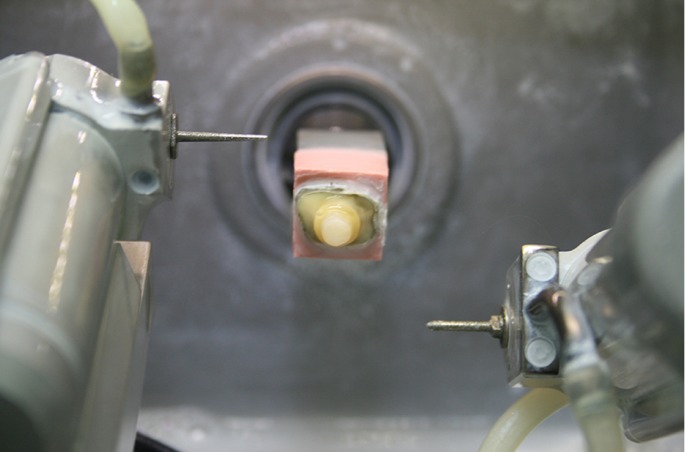
Milling a natural tooth to the virtual stump design.

After verifying the virtual stump design, the command “mill” was chosen, selecting a Vita CAD- Temp multicolor CTM-40® (VITA, Bad Säckingen, Germany) block size of the correct length in order to visualize the position of the molar within the total block. Once the desired virtual position had been established, a cone bur 12 and Cylinder Pointed Bur® (Sirona Dental Systems Gmbh) were selected. Then the modified block bearing the natural tooth was placed in the milling machine, the safety shield was closed, and milling began. When underway, the machine identifies and calibrates the block size, registering its height and width to determine if this is an already worked block or a new one. In this case, it recognized the existing dimensions as if this was an already used block and milled accordingly. Each specimen took approximately 27 minutes to mill and to avoid the piece being sectioned at the base in accordance with the design of the master model, the process was halted 5 minutes before completion in all cases (Fig. 3).

When all ten specimens had been milled they were placed in 2.4 cm diameter, 3.5 cm height copper cylinders, using a parallelizer to which a silicon key was fixed to the rod in order to position the specimens, and set in plaster at identical vertical positions.

## Results

Adapting CAD-CAD technology for working natural teeth made it possible to obtain ten prepared teeth of identical dimensions, exact replicas of the original virtual stump model.

The specimens were then used in a trial of complete coverage CAD-CAM provisional restorations, which set out to evaluate the retention of five temporary cements (10). Each study group (n=10) used the same ten specimens to perform a total of 50 tensile tests; these used ten CAD-CAM provisional restorations bonded with each of the five temporary cements to the ten identically prepared molars.

## Discussion

The lack of literature on reproducible specimen fabrication, which the present study set out to address, together with the failure to establish standardized protocols for stump fabrication, make it impossible to compare the results of *in vitro* trials.

Studies involving extracted teeth or replicas make use of a tooth stump worked to an ideal shape but this is fabricated manually and so is not reproducible. This method uses a master model which is reproduced by taking silicon impressions and then casting specimens in an appropriate material. But the fabrication and reproduction techniques themselves are sensitive and subject to accumulative error, at both the impression-taking and casting stages. Moreover, this method cannot be transferred to another research situation, except by means of the original physical master model. CAD-CAM technology eliminates impression-taking thanks to the use of a scanner. But the samples themselves are produced by milling a block of some artificial material, rather than a natural tooth (11).

Kelly states that when stump specimens have greater rigidity (comparing metal and resin, for example), a restoration ceramic bonded to them will resist greater forces (1). This means that strength data obtained will depend partly on the material used to fabricate the stump specimen.

A study by Rego used standardized dental specimens. Teeth were prepared with a high-speed turbine coupled to a surveyor with articulated rod to standardize a 6o taper and 4 mm height; but it was not possible to obtain specimens of identical dimensions (area/volume) (12).

Other *in vitro* works have focused on the restorations under investigation but pay little attention to the stump to which they are applied for testing (13).

CAD-CAM technology makes it possible to fabricate standardized specimens by using methods that (according to several authors) provide a number of advantages over the use of hand-made models. Firstly, they can be replicated exactly. The method is economical as it does not require alloys of noble metals or other expensive materials. It permits different designs adapted to both complete and partial coverage restorations (inlays, onlays, overlays and endocrowns). In studies of temporary cements, the specimens can be reused (10) but of course this is not possible in studies of definitive cements or bonding techniques on teeth. When natural teeth are used, the researcher can carry out different bonding techniques, which notably influence the behavior of the materials under investigation (1,14). The wetness of the dentin, its thickness and tooth pulp pressure are influential variables in *in vitro* studies that attempt to imitate *in vivo* conditions (15). Lastly, thermocycling processes will be closer to *in vivo* ageing processes if the sample is natural rather than artificial.

Establishing a set of standard measurements for stump specimens would have a beneficial effect on research outcomes; area and volume could be calculated and integrated in the protocols of different *in vitro* assays. These measurements should appear in a consensus document or as ISO norms.

Within the limitations of this *in vitro* study, it may be concluded that: CAD-CAM technology permits the standardization of natural extracted tooth preparation for *in vitro* trials and provides a reproducible method that could be applied to diverse studies allowing comparison between studies, and reducing bias deriving from variations in sample fabrication.
